# Estimation of dynamic SNP-heritability with Bayesian Gaussian process models

**DOI:** 10.1093/bioinformatics/btaa199

**Published:** 2020-03-18

**Authors:** Arttu Arjas, Andreas Hauptmann, Mikko J Sillanpää

**Affiliations:** b1 Research Unit of Mathematical Sciences, University of Oulu, Oulu FI-90014, Finland; b2 Department of Computer Science, University College London, London WC1E 6BT, UK; b3 Infotech Oulu, University of Oulu, Oulu FI-90014, Finland

## Abstract

**Motivation:**

Improved DNA technology has made it practical to estimate single-nucleotide polymorphism (SNP)-heritability among distantly related individuals with unknown relationships. For growth- and development-related traits, it is meaningful to base SNP-heritability estimation on longitudinal data due to the time-dependency of the process. However, only few statistical methods have been developed so far for estimating dynamic SNP-heritability and quantifying its full uncertainty.

**Results:**

We introduce a completely tuning-free Bayesian Gaussian process (GP)-based approach for estimating dynamic variance components and heritability as their function. For parameter estimation, we use a modern Markov Chain Monte Carlo method which allows full uncertainty quantification. Several datasets are analysed and our results clearly illustrate that the 95% credible intervals of the proposed joint estimation method (which ‘borrows strength’ from adjacent time points) are significantly narrower than of a two-stage baseline method that first estimates the variance components at each time point independently and then performs smoothing. We compare the method with a random regression model using MTG2 and BLUPF90 software and quantitative measures indicate superior performance of our method. Results are presented for simulated and real data with up to 1000 time points. Finally, we demonstrate scalability of the proposed method for simulated data with tens of thousands of individuals.

**Availability and implementation:**

The C++ implementation dynBGP and simulated data are available in GitHub: https://github.com/aarjas/dynBGP. The programmes can be run in R. Real datasets are available in QTL archive: https://phenome.jax.org/centers/QTLA.

**Supplementary information:**

Supplementary data are available at *Bioinformatics* online.

## 1 Introduction

Heritability, the proportion of phenotypic variation attributable to genetic factors, is a fundamental parameter in population and quantitative genetics (Visscher *et al.*, 2008). The aim in narrow-sense heritability estimation is to separate the variance of the trait into additive genetic and environmental variance components such that their sum equals the total variance of the trait. Generally, heritability is a population-specific parameter which can be estimated either using (i) linear mixed model (LMM) techniques (Henderson, 1984) or with (ii) multi-locus association (MLA) approaches (Sillanpää, 2011). In LMMs, information of the (additive) genetic relationships between the individuals in the population must be available, which can be determined either from known pedigree or from genomic data [single-nucleotide polymorphism (SNP)], while in MLA models, heritability can be estimated from genomic data. In LMMs, the trait variation is assumed to be controlled by a high number of small effect genes (polygenic), whereas MLA models assume that there are only few major genes behind the trait variation. We note, that in case heritability is estimated from genomic data, it is called SNP-heritability, and we will from now on refer by heritability specifically to narrow-sense SNP-heritability.

It is well known that heritability of a trait may depend on measurement time, age, environmental conditions (like temperature) or size (Stinchcombe *et al.*, 2012). In all such cases, it is natural to consider and estimate heritability as a dynamic function. In particular, functional variation in heritability is motivated by the fact that environmental changes may affect the environmental variance, and genes that control traits can activate or deactivate (Bryois *et al.*, 2017), which then may affect the genetic variance. In general, this kind of dynamic modelling of biological processes is a fast-growing field thanks to modern data collection techniques, see e.g. Moore *et al.* (2013) and Li and Sillanpää (2015). However, the number of statistical methods and associated (easy-to-use and publicly available) software packages to estimate dynamic heritability is still limited. To discuss this further, let us first put available methods into context. For approaches in the multivariate LMM framework we have the following options.

**Fig. 1.**
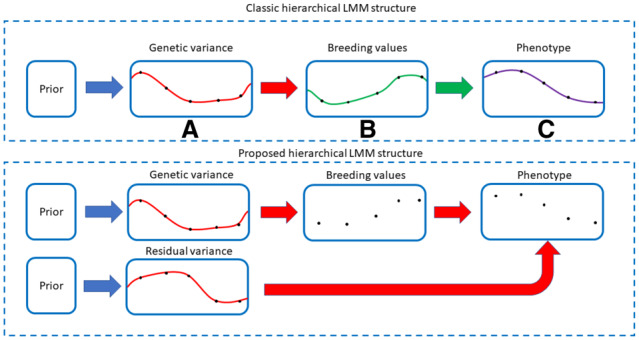
(Top panel) Continuous smoothing over time points can be induced into one of three possible layers in the LMM hierarchy: either in the variance component layer (**A**), genetic value layer (**B**) or phenotype layer (**C**). (Bottom panel) Our suggested LMM structure, where continuous smoothing over time points is induced on the genetic- and residual-variance components

- A simple approach: considers all time points as dependent traits and estimates their trait-specific genetic variances. This multi-trait model estimates variances jointly but does not apply any smoothing over time points (Henderson and Quaas, 1976; Lee and van der Werf, 2016).
- *Smoothing at phenotype level* (Fig. 1C): fits a linear or non-linear function over phenotypic time points, and then estimates latent-trait heritability influencing each parameter of that function using univariate or multivariate LMMs (e.g. Canaza-Cayo *et al.*, 2015).
- *Smoothing at breeding value level* (Fig. 1B): fits a linear or non-linear function over time in genomic breeding values (and possibly residual effects, i.e. permanent environmental effects). A common approach referred to as random regression (Campbell *et al.*, 2018; Schaeffer, 2016; http://animalbiosciences.uoguelph.ca/%7Elrs/BOOKS/rrmbook.pdf), also requires estimation of the residual covariance matrix if the permanent environmental effects are not smoothed.
- *Smoothing at variance component level* (Fig. 1A): if the residual variance is also smoothed, the residual covariance matrix can be left out of the model. Such an example is given by a spline-based method He *et al.* (2016, 2017) which is restricted to only twin data.

In comparison, for MLA approaches, we have the two possibilities.

- *Smoothing at phenotype level*: fits a linear or non-linear function over phenotypes, and then estimates latent-trait heritability for trend parameters (e.g. intercepts, slopes), by either using univariate or multivariate methods (see e.g. Gee *et al.*, 2003; Heuven and Janss, 2010; Li *et al.*, 2014; Sillanpää *et al.*, 2012).
- *Smoothing at quantitative trait locus (QTL) effect level*: fits a linear or non-linear function over time to QTL effects. In particular, these methods have been developed for QTL mapping of function-valued traits, but can also be applied for SNP-heritability estimation. Examples are varying-coefficient models, such as Li and Sillanpää (2013) and Vanhatalo *et al.* (2019). Considering the residual covariance structure in these models is recommended.

Dynamic modelling of biological phenomena can be beneficial for a number of reasons. For instance, by improving the precision of the estimation (Li and Sillanpää, 2013). This is due to the simple fact that, as with any statistical modelling, a larger dataset leads to increased precision, and naturally the amount of data increases with the number of measurement points. More importantly, if the nature of a phenomenon is dynamic, it is only rational to model the phenomenon dynamically and exploiting information content over time. Finally, there is a heated debate around heritability estimation as to why the current analysis methods are leading to a noticeable gap (known as missing heritability) between heritability estimates from SNP and pedigree/twin data (see e.g. Eichler *et al.*, 2010; Gibson, 2012; Young, 2019). One important contributor to this is the time-dependent nature of heritability, which our method aims to address.

One way to induce smoothing in the model (to model dynamic phenomena) is given by Gaussian processes (GPs). These are random processes whose degree of smoothness can be controlled by varying a set of parameters. In particular, they are appealing because of their analytical properties: with certain conjugacy structure the end result is given as an easy-to-calculate formula (Rasmussen and Williams, 2006). Furthermore, GPs have been successfully utilized in genetics before, e.g. by Vanhatalo *et al.* (2019), where associations between molecular markers and function-valued traits were studied. Also, the idea of using GPs in modelling dynamic biological processes is not new. For example, Pletcher and Geyer (1999) and Jaffrézic and Pletcher (2000) suggested that the genetic breeding values and environmental terms of the phenotype in LMM could be viewed as GPs. They also considered multivariate phenotypes by modelling the covariance function of a process through a parametric representation, which reduces the number of parameters in the model. In fact, this approach differs from ours, because we view the variance components of the LMM as GPs, which is similar to He *et al.* (2016, 2017), where variance processes were modelled with splines.

In this article, we specifically consider the Bayesian framework of statistical modelling. This means that the statistical inference is not only based on the measurement data and the statistical model but also on prior assumptions and information about the subject (Gelman *et al.*, 2013). For example, a common assumption in many research fields about dynamic processes is given by their smoothness, i.e. values at neighbouring time points are expected to be closer than at time points further apart. To estimate the parameters in Bayesian models, it is common to use Markov Chain Monte Carlo (MCMC) simulation methods (Robert and Casella, 2009), but since GP models usually have a high number of highly correlated parameters, efficient MCMC sampling is difficult. Thus, in addition to the traditional Metropolis–Hastings (MH) algorithm, we use the state-of-the-art method of elliptical slice sampling (Murray *et al.*, 2010), which has shown to perform well in GP models.

In addition to dynamic heritability estimation, the aim of this article is to illustrate the difference in the uncertainty of the estimates of joint and independent modelling of dependent traits. If a trait is measured longitudinally over time, the different measurements can be thought as individual traits. If there is dependence between traits, it makes sense to model them jointly. This dependence can be viewed as increased sample size because the value of one trait affects the value of the other.

This article is organized as follows: In Section 2, we present our proposed model for dynamic heritability estimation based on GPs. In addition, we introduce a two-stage method that is used as a baseline. In Section 3, we evaluate our method with two simulated and two real datasets and compare with a random regression model (RRM) implemented with MTG2 (Lee and van der Werf, 2016) and BLUPF90 (Misztal *et al.*, 2002) software and ACEt R-package for estimating dynamic heritability (He *et al.*, 2017). In Section 4, we examine the obtained results, followed by a discussion in Section 5.

## 2 Materials and methods

We will address the dynamic estimation of heritability by two methods, a joint estimation and a separated two-stage approach which serves to illustrate why joint modelling is beneficial. Specifically, the two-stage method first estimates the posterior means of the variance components and their credible intervals at every time point individually and subsequently combines the obtained estimates into smooth curves.

All models in this study are extensions of the basic LMM, defined as (Henderson, 1984).

(1) y=Xβ+Zu+ϵ,

where $y\in {\mathbb{R}}^{N}$ is a measurement vector, ***X*** is a matrix of fixed effects, β contains the regression coefficients associated with them and *N* is the number of individuals. The matrix ***Z*** (in this case Z=I) connects random effects $u\in {\mathbb{R}}^{N}∼N(0,\sigma_{G}^{2}G)$ with correct individuals. Here, $\sigma_{G}^{2}$ is the genetic variance and ***G*** is the genomic relationship matrix (defined in detail below). Lastly, $\epsilon \in {\mathbb{R}}^{N}∼N(0,\sigma_{E}^{2}I)$ is the error term. Since we do not have any fixed effects, we can express the centred measurement vector as $y_{c}=u+\epsilon$. We note that the overall covariance matrix of $y_{c}$ can be written as $K=\sigma_{G}^{2}G+\sigma_{E}^{2}I$. Narrow-sense heritability is then defined as $h^{2}=\sigma_{G}^{2}/(\sigma_{G}^{2}+\sigma_{E}^{2})$.

### 2.1 Genomic relationship matrix

To separate the genetic and environmental variance components, knowledge of the genetic relations between individuals is needed. A genomic relationship matrix can be estimated from molecular marker data. VanRaden (2008) describes the procedure of constructing such a matrix. First, let ***M*** be an *N *×* M* genotype matrix where *N* is the number of individuals and *M* is the number of markers. The elements in ***M*** are coded as −1,0,1 for homozygote, heterozygote and the other homozygote, respectively. Second, let *p_i_* be the allele frequency of the second allele at locus *i*. Then, we construct a matrix ***P*** with the same dimensions as ***M*** and set the *i*th column of ***P*** to $2(p_{i}-0.5)$. Let Q=M−P. Finally, we can construct a genomic relationship matrix as:

(2) $$G=\frac{QQ\prime }{2\sum_{i}p_{i}(1-p_{i})}.$$

To reduce the estimation error, we used a shrinkage estimated version of this matrix, defined in detail by Endelman and Jannink (2012) and implemented in the R-package rrBLUP (Endelman, 2011). We note that the estimated genomic relationship matrix ***G*** is positive-semidefinite and hence might not be invertible. However, for our model formulation, this will not be a problem. This is due to the fact that we always add positive values to the diagonal of the matrix; see matrix **K **above.

### 2.2 Joint model over time points

To extend the model (1) to the case of longitudinal data, we can use the Kronecker product denoted by ⊗. The extended model defines a probability distribution for a data vector ${\tilde{\text{y}}}_{c}=[y_{c}(1)\ldots y_{c}(T)]\prime$ that can be written as

(3) $${\tilde{y}}_{c}=\tilde{u}+\tilde{\epsilon },\;\;\;\;{\tilde{y}}_{c},\tilde{u},\tilde{\epsilon }\in {\mathbb{R}}^{NT}$$

where $\tilde{u}=[u(1)\ldots u(T)]\prime ∼N(0,\text{diag}(\sigma_{G}^{2}(1),\ldots ,\sigma_{G}^{2}(T))⊗G)$ contains all breeding values and $\tilde{\epsilon }=[\epsilon (1)\ldots \epsilon (T)]\prime \sim N(0,\text{diag}(\sigma_{E}^{2}(1),\ldots ,\sigma_{E}^{2}(T))⊗I)$ contains all error terms from individual time points consecutively and *T* is the number of time points. The overall covariance matrix for ${\tilde{y}}_{c}$ can be written as $\tilde{\text{K}}=\text{diag}(\sigma_{G}^{2}(1),\ldots ,\sigma_{G}^{2}(T))⊗G+\text{diag}(\sigma_{E}^{2}(1),\ldots ,\sigma_{E}^{2}(T))⊗I$. The variance component vectors can now be estimated with a Bayesian approach by setting suitable priors for them. It is worth noting that this model structure implies that all covariances across time (within and between individuals) are ignored. For the rapid performance of the algorithm, this assumption is crucial. It is in the priors of the variance component vectors where we assume dependence over time (see Fig. 1, bottom panel). This is a fundamental difference compared with for example Pletcher and Geyer (1999) who model the dependence on the level of breeding values. The idea behind our assumption is that the dependence of neighbouring trait values induces dependence on the variance level.

The priors are based on assumptions about the qualitative features of the variance component functions, namely their continuity and smoothness. Due to the aforementioned reasons, we assume that the variance values at neighbouring time points are on average closer than at time points further apart. To formulate such assumptions mathematically, one can use GPs, fully defined by a mean function and a covariance function (Rasmussen and Williams, 2006). We write $\text{log}\;\sigma_{G}^{2}∼GP(0,C_{G}(t,t\prime ))$ and $\text{log}\;\sigma_{E}^{2}∼GP(0,C_{E}(t,t\prime ))$, where $C_{G}(t,t\prime )=\text{cov}(\text{log}\;\sigma_{G}^{2}(t),\;\text{log}\;\sigma_{G}^{2}(t\prime ))$ and $C_{E}(t,t\prime )=\text{cov}(\text{log}\;\sigma_{E}^{2}(t),\;\text{log}\;\sigma_{E}^{2}(t\prime ))$. Logarithms are used to allow negative values for the processes and the zero mean can be achieved approximately by suitable translation of the data. For *C_G_* and *C_E_*, we use the Matérn covariance function, given by

(4) $$C(t,t\prime )=\tau^{2}\frac{2^{1-\nu }}{\Gamma (\nu )}{\left(\frac{\left|t-t^{\prime }\right|}{\lambda }\right)}^{\nu }K_{\nu }\left(\frac{\left|t-t\prime \right|}{\lambda }\right),$$

where $\tau^{2}$ is the magnitude parameter that controls the overall variance of the stochastic process, *λ* is the length scale that governs how fast the covariance drops with respect to the distance of time points, Γ(·) denotes the gamma function and $K_{\nu }(\cdot )$ the modified Bessel function of the second kind. The value of *ν* controls the mean-square differentiability of the process, which affects its smoothness. We note that for ν=0.5,1.5,2.5,…, there exists a simple form for the covariance function. In this study, we fix ν=1.5, which means that a sample process is once mean-square differentiable and we obtain

(5) $$C_{1.5}(t,t\prime )=\tau^{2}\left(1+\frac{\left|t-t\prime \right|}{\lambda }\right)\text{exp}\;(-\frac{\left|t-t\prime \right|}{\lambda }).$$

This means the process is rather smooth, but it is also allowed to change rapidly (Rasmussen and Williams, 2006).

A common problem with GPs is determining values for the hyperparameters $\tau^{2}$ and *λ*. In the Bayesian framework, one can set up separate priors for each and estimate them together with the other unknowns using MCMC. However, it is known that identifiability issues arise when simultaneously estimating both hyperparameters (Zhang, 2004). Thus, it is common to fix one of them. In our case, we fix $\tau^{2}=1$ and scale the data so that the mean of the overall variance over time points equals 2. This means the mean of the environmental and genetic variance over time points is 1 if they are equal.

Because our GP model is non-parametric, we have to discretize the time axis and define a grid of points where we estimate the variance components. For simplicity, we estimate the variance components at the same locations the measurements were taken. This means that the covariance function C(·,·) becomes a covariance matrix ***C***. Then we have for each element in the matrix that ${\left[C\right]}_{ij}:=C(t_{i},t_{j})$. We note that a covariance matrix defined this way is dense and computationally heavy to operate with. Hence, we use a sparse approximation for the inverse of the covariance matrix (Roininen *et al.*, 2014), discussed in more detail in the Supplementary Material.

The model can be written in hierarchical form as

(6) $$\begin{array}{cc} {\tilde{\text{y}}}_{c}|\sigma_{E}^{2},\sigma_{G}^{2} & \sim N(0,\tilde{\text{K}}),\\ \;\text{log}\;\sigma_{E}^{2}|\lambda_{E} & \sim N(0,C_{E}),\\ \;\text{log}\;\sigma_{G}^{2}|\lambda_{G} & \sim N(0,C_{G}),\\ \;\text{log}\;\lambda_{E} & \sim N(\mu_{\lambda },\zeta_{\lambda }^{2}),\\ \;\text{log}\;\lambda_{G} & \sim N(\mu_{\lambda },\zeta_{\lambda }^{2}). \end{array}$$

By Bayes’ formula, the unnormalized posterior density can be expressed as

$$\begin{array}{c} p(\sigma_{G}^{2},\sigma_{E}^{2},\lambda_{E},\lambda_{G}|{\tilde{\text{y}}}_{c})\propto \\ p_{N}({\tilde{\text{y}}}_{c}|0,\tilde{\text{K}})p_{N}(\text{log}\;\sigma_{G}^{2}|0,C_{G})p_{N}(\text{log}\;\sigma_{E}^{2}|0,C_{E})\\ p_{N}(\text{log}\;\lambda_{E}|\mu_{\lambda },\zeta_{\lambda }^{2})p_{N}(\text{log}\;\lambda_{G}|\mu_{\lambda },\zeta_{\lambda }^{2}), \end{array}$$

where $p_{N}$ refers to the multinormal density.

In practice, with this parameterization, we observed poor mixing of the hyperparameter chains. Hence, we employed whitening (Monterrubio-Gómez *et al.*, 2020; Murray and Adams, 2010; Yu and Meng, 2011), which breaks the dependencies of the variance component processes and corresponding hyperparameters under the prior. We first note that the logarithmized variance component processes can be expressed as $\text{log}\;\sigma_{E}^{2}=C_{E}^{\frac{1}{2}}\eta_{E}$ and $\text{log}\;\sigma_{G}^{2}=C_{G}^{\frac{1}{2}}\eta_{G}$, where $\eta_{E}$ and $\eta_{G}$ are both standard multivariate normally distributed. The matrix square roots are obtained straightforwardly through the approximations (see Supplementary Material). Instead of $\text{log}\;\sigma_{E}^{2}$ and $\text{log}\;\sigma_{G}^{2}$, we now sample $\eta_{E}$ and $\eta_{G}$. The reparametrization corresponds to the following posterior density

$$\begin{array}{c} p(\eta_{G},\eta_{E},\lambda_{E},\lambda_{G}|{\tilde{\text{y}}}_{c})\propto \;p_{N}({\tilde{\text{y}}}_{c}|0,\tilde{\text{K}})p_{N}(\eta_{G}|0,I)p_{N}(\eta_{E}|0,I)\\ p_{N}(\text{log}\;\lambda_{E}|\mu_{\lambda },\zeta_{\lambda }^{2})p_{N}(\text{log}\;\lambda_{G}|\mu_{\lambda },\zeta_{\lambda }^{2}), \end{array}$$

where

$$\tilde{K}=\text{diag}\left(\text{exp}\;\left\{C_{G}^{\frac{1}{2}}\eta_{G}\right\}\right)⊗G+\text{diag}\left(\text{exp}\;\left\{C_{E}^{\frac{1}{2}}\eta_{E}\right\}\right)⊗I.$$

The hyperparameters $\mu_{\lambda }$ and $\zeta_{\lambda }^{2}$ are set by following Monterrubio-Gómez *et al.* (2020). In particular, it is based on the idea that the length scale is identifiable between the smallest and largest distance between two time points, say [a,b]. Hence we want to place most of its prior probability mass in that interval. By using the quantile function of a standard normal distribution, we can assign ∼95% of the prior mass between the interval by solving the following system of equations:

(7) $$\begin{array}{cc} \mu_{\lambda }-1.96\zeta_{\lambda } & =\text{log}\;a\\ \mu_{\lambda }+1.96\zeta_{\lambda } & =\text{log}\;b. \end{array}$$

We emphasize that this model specification leaves no tuning parameters to fix prior to estimation which eliminates the need for preliminary analyses and consequently saves plenty of time.

#### 2.2.1 Parameter estimation in the joint model

To generate dependent MCMC samples from the posterior distribution of parameters $\eta_{G}$ and $\eta_{E}$, we use the elliptical slice sampling method (Murray *et al.*, 2010). It is a rejection-free sampling algorithm and we noticed that it does perform better for high-dimensional data (more than 100 time points) than the block-update of MH algorithm. The problem with elliptical slice sampling is that the likelihood must be evaluated multiple times in a single MCMC iteration, making it quite a bit slower than MH. For the length scales, we use MH with Gaussian random walk proposals, i.e. the proposal value is sampled from the distribution $N(\theta^{\left(i\right)},\sigma_{\text{RW}}^{2})$, where $\theta^{\left(i\right)}$ is the current value of the parameter. The variance is adapted following Section 3 in Roberts and Rosenthal (2009) to achieve an acceptance rate of 0.44 which is considered optimal in certain settings. The four parameter subsets ($\eta_{E},\eta_{G},\;\text{log}\;\lambda_{E},\;\text{log}\;\lambda_{G}$) are sampled in alternatingly while keeping the other values fixed.

The computationally most intensive part of the algorithm is the evaluation of the likelihood function, due to the inversion and determinant computation of an *NT* × *NT* covariance matrix. Fortunately, the form of the matrix allows us to utilize some basic linear algebra to make the computations feasible. We start by examining the structure of matrix $\tilde{\text{K}}$ and note the block diagonal structure where the blocks consist of time point-specific covariance matrices:

$$\tilde{\text{K}}=\left[\begin{array}{cccc} \sigma_{G}^{2}(1)G+\sigma_{E}^{2}(1)I & 0 & \ldots & 0\\ \vdots & \vdots & \ddots & \vdots \\ 0 & 0 & \ldots & \sigma_{G}^{2}(T)G+\sigma_{E}^{2}(T)I \end{array}\right].$$

This means, we can express the overall log-likelihood function of the parameters as a sum of log-likelihood functions of each time point

(8) $$\text{log}\;p({\tilde{\text{y}}}_{c}|0,\tilde{\text{K}})=\sum_{t=1}^{T}\;\text{log}\;p(y_{c}(t)|0,K(t)).$$

Similarly to Kang* et al.* (2008), we can now use eigen decomposition to decorrelate the measurements using a linear transformation to speed up the computations. The covariance matrix of the measurements y(t) at time *t* can be decomposed as UD(t)U′, where ***U*** is the eigenvector matrix of ***G*** and D(t) is a diagonal matrix with ${\left[D(t)\right]}_{nn}=\sigma_{G}^{2}(t)\xi_{n}+\sigma_{E}^{2}(t)$ and *ξ_n_* being the eigenvalues of ***G***. By the orthogonality of ***U***, we have that var(U′y(t))=U′UD(t)U′U=D(t), implicating that the elements in the transformed measurement vector U′y(t) are independent of each other. This reduces the log-likelihood calculation into a sum. Most importantly, since ***U*** does not depend on the variance components, the transformation has to be done only once. Setting z(t)=U′y(t), the resulting log-likelihood can be written as

(9) $$\text{log}\;p(\tilde{\text{y}}|0,\tilde{\text{K}})=\sum_{t=1}^{T}\sum_{n=1}^{N}\;\text{log}\;p(z{\left(t\right)}_{n}|0,\sigma_{G}^{2}(t)\xi_{n}+\sigma_{E}^{2}(t)).$$

The pseudocode for the algorithm can be found in the Supplementary Algorithm S1.

### 2.3 Two-stage method

To illustrate the benefits of the joint model, we compare it to a baseline approach. The method consists of two stages: (i) the estimation of posterior means of the variance components and their 95% credible intervals at each time point separately and (ii) combining individual estimates together by smoothing the obtained estimates from stage one. The estimation in stage one is based on the same model as in the joint estimation but for one time point

(10) $$y_{c}(t)=u(t)+\epsilon (t),$$

where $y_{c}$ is the centred measurement vector at time *t*, $u(t)∼N(0,\sigma_{G}^{2}(t)G)$ and $\epsilon (t)∼N(0,\sigma_{E}^{2}(t)I)$. As in the joint method, the data are scaled such that the overall variance over time points is 2. The covariance matrix of $y_{c}(t)$ is $K(t)=\sigma_{G}^{2}(t)G+\sigma_{E}^{2}(t)I$ and the model can be written as

(11) $$\begin{array}{cc} y_{c}(t) & ∼N(0,K(t)),\\ \;\text{log}\;\sigma_{G}^{2}(t) & ∼N(0,1),\\ \;\text{log}\;\sigma_{E}^{2}(t) & ∼N(0,1). \end{array}$$

We note that this is a special case of the joint model presented earlier, see Equation (3). Posterior means and 95% credible intervals of the variance components at each time point are obtained from this analysis used in the next stage.

Smoothing of the variance component curves over time is based on the model

(12) $$y_{\sigma^{2}}=f_{\sigma^{2}}+\epsilon_{\sigma^{2}},$$

where $y_{\sigma^{2}}$ contains either logarithmized posterior means, lower 95% credible interval limits or upper 95% credible interval limits estimated in stage one, $f_{\sigma^{2}}∼GP(0,C_{\lambda }(t,t\prime ))$ is a smooth process and $\epsilon_{\sigma^{2}}\sim N(0,\gamma^{2}I)$ is an error term. $C_{\lambda }(\cdot ,\cdot )$ is again the Matérn covariance function, defined in Equation (5). Here, $y_{\sigma^{2}}$ is scaled to have mean zero and variance one. After discretization, the model can be written as follows:

(13) $$\begin{array}{cc} y_{\sigma^{2}} & ∼N(0,C_{\lambda }+\gamma^{2}I),\\ \;\text{log}\;\gamma^{2} & ∼N(0,10000),\\ \;\text{log}\;\lambda & ∼N(\mu_{\lambda },\zeta_{\lambda }^{2}), \end{array}$$

where $\gamma^{2}$ and *λ* are assumed to be a priori independent. The variance parameter in the error term is given an uninformative prior and the parameters $\mu_{\lambda }$ and $\zeta_{\lambda }^{2}$ are fixed similarly as in joint estimation method following Monterrubio-Gómez *et al.* (2020). The process $f_{\sigma^{2}}$ is analytically integrated out of the model, but we can reconstruct it at the measurement points after the estimation of the hyperparameters (Rasmussen and Williams, 2006) by noting that

$$\begin{array}{c} f_{\sigma^{2}}|y_{\sigma^{2}},\lambda ,\gamma^{2}\sim \\ N(C_{\lambda }{\left(C_{\lambda }+\gamma^{2}I\right)}^{-1}y_{\sigma^{2}},C_{\lambda }-C_{\lambda }{\left(C_{\lambda }+\gamma^{2}I\right)}^{-1}C_{\lambda }). \end{array}$$

#### 2.3.1 Parameter estimation in the two-stage method

The parameters $\text{log}\;\sigma_{G}^{2}(t)$ and $\text{log}\;\sigma_{E}^{2}(t)$ in stage one are estimated using a random walk MH algorithm with the same adaptation as in the joint estimation method. They are sampled at MCMC iteration *i *+* *1 from the distributions $N(\text{log}\;\sigma_{G}^{2}{\left(t\right)}^{\left(i\right)},s_{\sigma_{G}^{2}}{\left(t\right)}^{\left(i\right)})\;\;\text{and}\;\;N(\text{log}\;\sigma_{E}^{2}{\left(t\right)}^{\left(i\right)},s_{\sigma_{E}^{2}}{\left(t\right)}^{\left(i\right)})$. The proposals are accepted by MH (a single parameter at a time) conditionally on the other parameter fixed to its latest value.

The parameters $\text{log}\;\gamma^{2}\;\;\text{and}\;\;\;\text{log}\;\lambda$ of the smoothing model in phase two are estimated similarly as in stage one with the same adaptation. They are sampled at iteration i+1 from the distributions $N(\text{log}\;\gamma^{2(i)},s_{\gamma^{2}}^{\left(i\right)})\;\;\text{and}\;\;N(\text{log}\;\lambda^{\left(i\right)},s_{\lambda }^{\left(i\right)})$. The proposals are accepted by MH (a single parameter at a time) conditionally on the other parameter fixed to its latest value.

The pseudocode for both stages can be found in the Supplementary Algorithms S2 and S3.

### 2.4 Computational considerations

Since the log-likelihood in the joint estimation method can be expressed as a sum (Equation 9), its computation can be parallelized. We parallelized all log-likelihood calculations, gaining additional speedups. The computation times were between 24 min for the mouse activity data and 31 min for the *Arabidopsis* data with 300 000 MCMC iterations. The workstation used for the simulations had an AMD Ryzen Threadripper 2950X 3.5 GHz processor with 16 cores and 32 GB of RAM. The method is implemented with C++ integrated with R using the Rcpp library (Eddelbuettel and François, 2011) and Eigen (Guennebaud *et al.*, 2010) (http://eigen.tuxfamily.org) for linear algebra. The programme is available at https://github.com/aarjas/dynBGP.

## 3 Example analyses

To test the methods described earlier, we used four different datasets with a large number of time points. Two were simulated and the two others were real datasets from https://phenome.jax.org/centers/QTLA.

### 3.1 Simulated dataset

The simulated data consist of measurements from N=1000 individuals from T=50 time points. First, a relationship matrix was created by generating an N×N matrix  S with independent standard normally distributed elements. The relationship matrix was then computed as $G=SS^{T}/N+0.1I$. A small number was added to the diagonal to make the matrix positive definite. To simulate realistic data, the longitudinal dependencies need be taken into account as well. This was done by computing a T×T GP matrix C where the row i and column j intersection was set to ${\left[C\right]}_{ij}=\left(1+\frac{\sqrt{5}|t_{i}-t_{j}|}{\left(50/3\right)}+\frac{5{\left(t_{i}-t_{j}\right)}^{2}}{3\cdot {\left(50/3\right)}^{2}}\right)\text{exp}\;\left(-\frac{\sqrt{5}|t_{i}-t_{j}|}{\left(50/3\right)}\right)$. The genetic and environmental components of the data were simulated from the distributions N(0,C⊗G)  and  N(0,C⊗I), respectively. Finally, the components were scaled with the corresponding variances at each time point and summed. A correct relationship matrix was assumed known in the analysis stage. To demonstrate scalability of the approach, we created larger datasets with up to 50 000 individuals and 1000 time points (see Supplementary Fig. S5).

### 3.2 *Arabidopsis thaliana* dataset

The second dataset contains *A.thaliana* root tip angle measurements (Moore *et al.*, 2013). The population consists of N=162 recombinant inbred lines of *Arabidopsis* seeds with 234 markers. The seeds were placed on Petri dishes that were held in front of a camera and rotated 90° so that the roots grew parallel to the ground. The root tip angle was measured every 2 min for 8 h resulting in T=239 time points. Moore *et al.* (2013) describe the measurement process in more detail. We used a version of the data where the phenotype values were averages of multiple individuals representing the same line.

### 3.3 Mouse activity dataset

The third dataset consists of mouse activity measurements (Xiong *et al.*, 2011). The activity of N=89 mouses was monitored over a period of 12 days. One day was divided into 6-min intervals and an active state probability of a given mouse in each interval was calculated based on the 12 days of data. This results in T=222 time points. Since the outcome is a probability, normality assumption is not realistic. Hence, we transformed the phenotypic data using logit-transformation following the procedure described by Li and Sillanpää (2013). The data also have measurements of 251 markers.

### 3.4 Comparison with ACEt

We also compared our joint estimation method with the freely available ACEt R-package for estimating dynamic heritability (He *et al.*, 2016, 2017). The method is restricted only for twin data and it uses splines in defining the different dynamic variance components. The ACEt model also includes a common environmental effect as a third variance component, which our model lacks. For uncertainty quantification, ACEt uses delta-method or bootstrap. For the method comparison, we used a simulated twin dataset that comes with the ACEt R-package. It consists of 100 twin pairs, half of who are monozygotic and half of who are dizygotic. The N=200 individuals have T=50 equispaced measurements of an artificial trait over 50 years. In twin models, the covariances in the relationship matrix between a monozygotic twin pair and a dizygotic twin pair are assumed to be 1 and 0.5, respectively, while the diagonal values are all 1. Between-pair covariance is assumed to be 0.

### 3.5 Completing missing genotype data by imputation

There were no missing phenotype values in any of the datasets. The mouse activity dataset had 0.3% and the *Arabidopsis* dataset 1.4% missing marker values. All missing values were imputed once before calculating the genomic relationship matrix with the mean value of the given marker over individuals.

## 4 Results

**Fig. 2.**
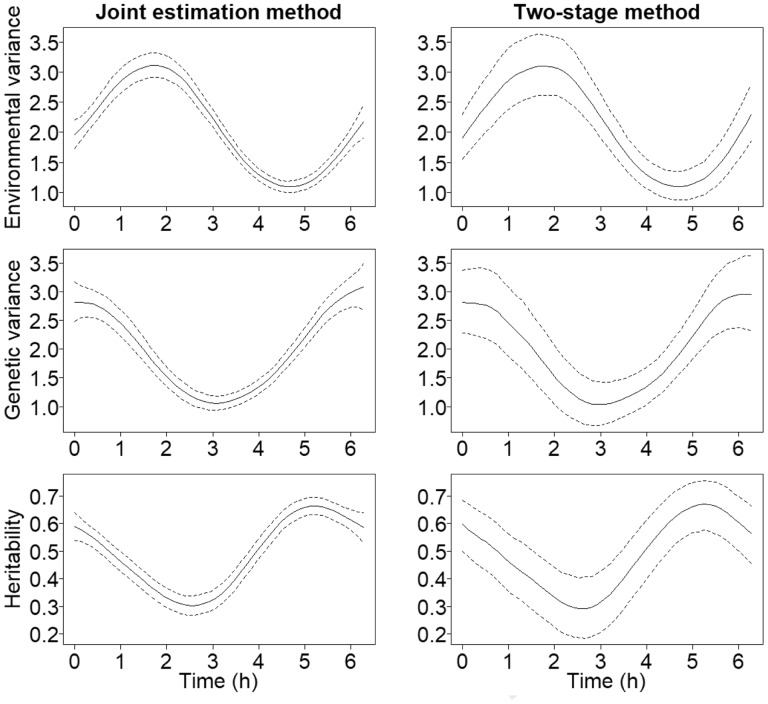
The dynamic variance components and SNP-heritability estimated from the simulated dataset with the two different Bayesian methods. The posterior mean curves are drawn with solid lines and 95% credible intervals with dashed lines

The results from the Bayesian analysis of simulated data are presented in Figure 2 for both methods. The posterior estimated variance component functions generally follow the real functions that were used to generate the data well. Perhaps the most interesting aspect about the results is the difference in the uncertainty of the estimates between the methods. The 95% credible intervals provided by the joint model are far narrower than those of the two-stage method. This is because the joint model makes use of the whole dataset, whereas the two-stage method uses the data of each time point independently.

**Fig. 3.**
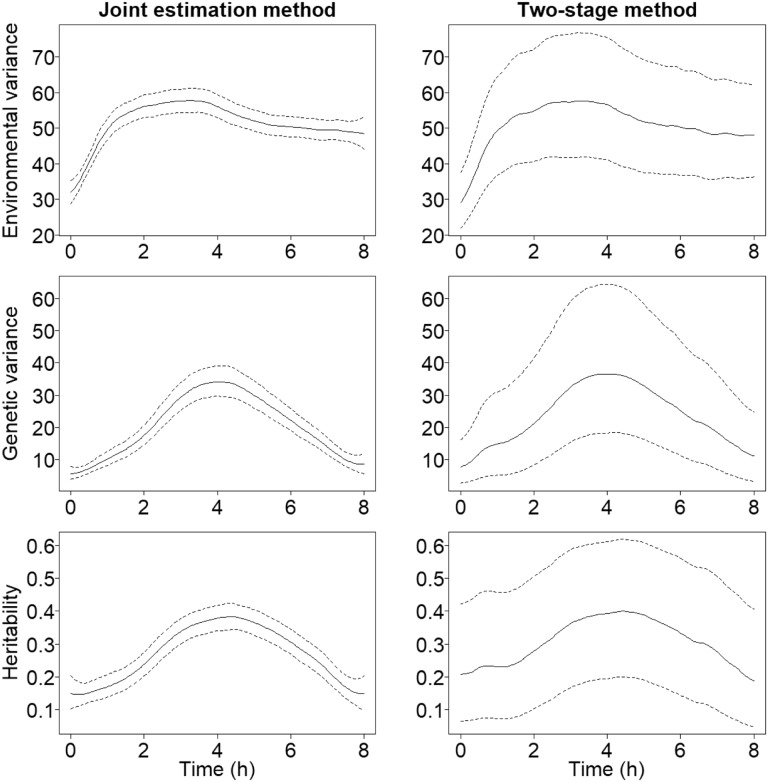
The dynamic variance components and SNP-heritability estimated from the *A.thaliana* dataset with the two different Bayesian methods. Posterior mean curves are drawn with solid lines and 95% credible intervals with dashed lines

The estimated posterior mean curves and their 95% credible intervals for variance components and SNP-heritability calculated from the *Arabidopsis* seed data are presented for the joint model and two-stage method in Figure 3. Again, one can clearly see much wider 95% credible intervals surrounding the posterior mean curves obtained from the two-stage method. Moore *et al.* (2013) have also estimated the dynamic heritability from this dataset using ANOVA. They calculated the variances within and between genetically distinct lines separately at every point in time. The curve has the same shape but the actual values are somewhat smaller than ours, peaking at 0.25, while our estimate peaks at about 0.4. This offset can be explained by different averaging of used data and in particular, their estimation lacks uncertainty estimates. Vanhatalo *et al.* (2019) have analysed the same data as well and are likely using the same averaging as we do. We note that their obtained heritability estimates coincide very well with ours. However, they also lack uncertainty estimates. Interestingly, similar results were not expected, since the two analysis models differ significantly in structure and especially in terms of assumptions on the underlying genetics.

**Fig. 4.**
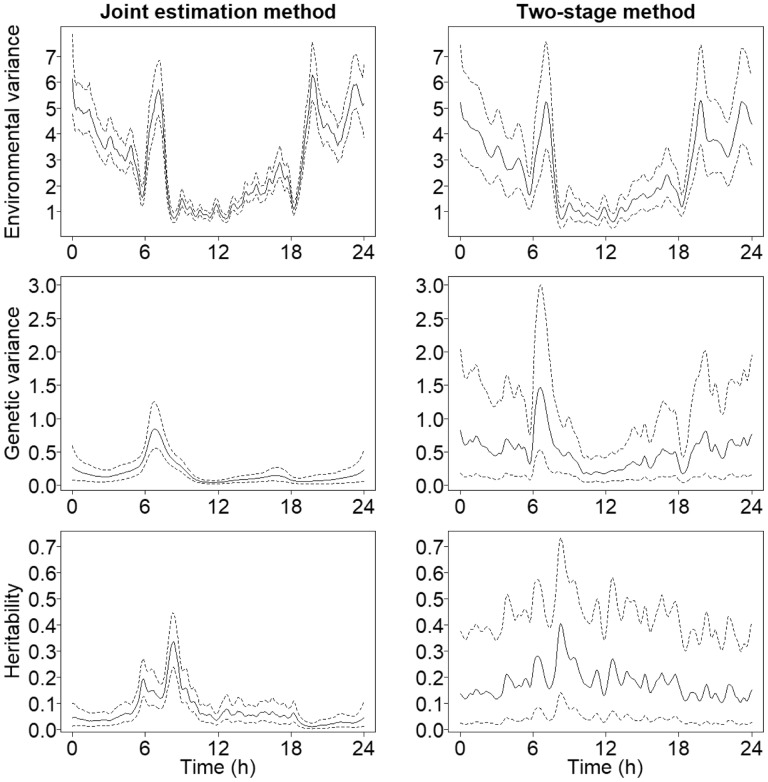
The dynamic variance components and SNP-heritability estimated from the mouse activity dataset with the two different Bayesian methods. Posterior mean curves are drawn with solid lines and 95% credible intervals with dashed lines

The posterior estimates from the mouse activity data are presented in Figure 4. This kind of data seems to be challenging for the GP models. This is mainly due to the fact that here the variance processes have very rough features along with smooth areas. Thus, the amount of smoothing needed is very different at different time points. Especially, the heritability process estimated with the two-stage method does not perform so well and produces highly oscillating features. The same data were analysed in Vanhatalo *et al.* (2019) as well. In this case, their heritability estimates look quite different to ours. In particular, our methods have smoothed most rough edges, whereas their estimate has preserved them. Nevertheless, the overall shape is similar. These differences here are likely the consequence of using very different models.

We also compared the joint model with a RRM [implemented in e.g. BLUPF90 (Misztal *et al.*, 2002), MCMCglmm (Hadfield, 2010) and MTG2 (Lee and van der Werf, 2016)] which is a well-established method for analyzing dynamic biological phenomena. We simulated 10 different datasets with the method from Section 3.1 and fitted a Legendre polynomial of degree five for both the genomic breeding values and permanent environmental effects. The model assumes heterogenous residual variances over time. A more precise model definition can be found in the Supplementary Material. We computed the mean squared error (MSE) of the estimated genetic and environmental variances as well as heritabilities, where $\text{MSE}=(1/T)\sum_{t=1}^{T}\left(f(t\right)-\hat{f}(t){)}^{2}$, with f(t) being the ground truth at time $t\;\text{and}\;\hat{f}(t)$ the estimate at time t. We used posterior mean as the point estimate for the joint method. The computed errors can be found in the Supplementary Table S1. This analysis was performed using AI-REML method in the software MTG2 (Lee and van der Werf, 2016) and it took on average 14 min. The MSE of the joint method was on average 17% smaller than of the RRM. We note that the MSE varies with the chosen degree of the Legendre polynomials and for this computation; we chose the best performing combinations with a reasonable computation time. Additionally, we fitted a Bayesian version of the same model using BLUPF90 family of programmes (Misztal *et al.*, 2002) and GIBBS2F90 in particular. This allowed us to quantify the uncertainty in the variance components estimated with the RRM. The results of the analysis and further details can be found in the Supplementary Figure S7. The 95% credible intervals obtained from the RRM are slightly wider than of the joint model. This might be due to the differences in model structures (cf. Fig. 1). The computation time for the Bayesian RRM for the simulated dataset was over 24 h, whereas for the joint method it was <30 min with 300 000 MCMC-iterations in both. Furthermore, we would like to mention that the RRM implemented in both MTG2 and GIBBS2F90 did not converge on our real dataset examples where the number of individuals is small but the number of time points high. Yet, the joint method manages to do so, i.e. the MCMC sampler converges. It is not completely clear to us why this is the case. One possibility might be the difference in assumptions: In RRMs the assumptions about the parametric shape of the breeding values and environmental effects induce some shape for the variance components. In the joint method, the prior assumptions concern only the variances. This direct modelling strategy might simplify the estimation process.

Finally, the comparison of our joint model with the ACEt model for analyzing twin data is presented in the Supplementary Figure S4. The results obtained by both methods are consistent, even though ACEt considers also common environment in its model. In our method, the genetic variance component seems to have absorbed the common environmental variation, resulting in slightly higher heritability estimate than the one given by ACEt. The uncertainty estimates differ a bit between the methods, which is likely due to the common environmental component. Moreover, obtained uncertainty limits are not fully comparable because the frequentist ACEt method uses delta-method and our Bayesian method uses MCMC sampling for generating the limits.

Performance of the MCMC-algorithm for the joint model can be evaluated from traceplots of the parameters (Supplementary Figs S1–S3). All of the parameter chains seem to have converged, although there is some oscillation on the variance component parameters with the lowest effective sample size.

## 5 Discussion and conclusions

We presented a new Bayesian method for estimating dynamic narrow-sense heritability, based on LMMs and GP priors. The method uses data from all time points at once, making it possible for the time points to ‘borrow strength’ from one another through the prior covariance structure. This property makes the resulting posterior distributions narrower compared with the second method where the variance components are estimated separately for each time point and smoothed afterwards. Another benefit of the method presented due to non-parametric smoothing using GPs is that it can handle very general functional shapes.

The presented estimation method bears some similarities with random regression as both are based on LMMs, but there are essential differences that we would like to point out. Most notably, in our proposed method the smoothing is based on priors that are set for the variance component vectors, while in random regression the smoothness of the variances is induced through the assumptions made about the functional shape of the breeding values and environmental effects. Additionally, our model assumes independence of different traits (measurements made at different points in time) and ties them together with the priors. In multivariate LMMs, traits are assumed to be dependent and random regression attempts to reduce the size of the estimated covariance matrix by reparametrization.

A benefit of our proposed method is the ability to quantify the uncertainty in the variance component estimates. This is also possible in Bayesian RRMs using MCMC but in frequentistic RRMs additional steps such as application of delta-method or bootstrap are needed to produce the respective confidence intervals. In addition, our method is completely free of tuning whereas in random regression the degree of the polynomial or alternatively knot points in splines must be chosen prior to estimation. In practice, we noticed that the estimation in MTG2 is really fast for a few traits but it slows down quickly as the number of traits increases. In fact, trying to run the algorithm with 1000 individuals and 100 traits causes an insufficient virtual memory error. In contrast, our algorithm still worked well with 50 000 individuals and 100 time points within a reasonable time (∼20 h). Additionally, high number of traits is not an issue either as demonstrated in the Supplementary Figure S5. We like to emphasize that the computation times grow almost linearly and hence our algorithm exhibits excellent scalability. Based on our results, we believe that random regression performs best when there is a high number of individuals and low number of time points which was not the case in our real data examples. In MTG2, the time complexity is cubic with respect to the number of time points (Lee and van der Werf, 2016), and we believe this is also the case with other RRM implementations. It is also noted by Schaeffer (2016) that Legendre polynomials might cause artefacts near the boundaries of the covariate domain and hence GPs are favoured. A limitation of our method is the inability to model gene-covariate interactions with possibly only one measurement per individual (cf. Moore *et al.*, 2019; Ni *et al.*, 2019). Theoretically, to perform such analysis, the covariate would have to be split up into discrete groups with each group containing suitably large population (cf. Robinson *et al.*, 2017). This would also result in each group having their own relationship matrix. This is not supported by the algorithm at the moment, however.

Furthermore, the method presented here can also be extended for further analysis. For example, given the posterior mean estimates ${\hat{\sigma }}_{G}^{2}(t)\;\;\text{and}\;\;{\hat{\sigma }}_{E}^{2}(t)$ at time t, the conditional distribution of genomic breeding values $u(t)\;\;\text{is}\;\;N(\mu_{u}(t),\Sigma_{u}(t))$ (Rasmussen and Williams, 2006), where

(14) $$\begin{array}{cc} \mu_{u}(t) & ={\hat{\sigma }}_{G}^{2}(t)G{\left({\hat{\sigma }}_{G}^{2}(t)G+{\hat{\sigma }}_{E}^{2}(t)I\right)}^{-1}y(t)\;\text{and}\\ \Sigma_{u}(t) & ={\hat{\sigma }}_{G}^{2}(t)G-{\hat{\sigma }}_{G}^{2}(t)G{\left({\hat{\sigma }}_{G}^{2}(t)G+{\hat{\sigma }}_{E}^{2}(t)I\right)}^{-1}{\hat{\sigma }}_{G}^{2}(t)G. \end{array}$$

The posterior means of genomic breeding values are not smooth functions over time (cf. Campbell *et al.*, 2018). After computing the breeding values, the SNP effects m(t) can be estimated for association mapping purposes by the back-transformation formula $\hat{m}(t)=R\prime G^{-1}\mu_{u}(t)$, where ${\left[R\right]}_{ij}=({\left[M\right]}_{ij}+1-2p_{j}){\left(\sqrt{2p_{j}(1-p_{j})}\right)}^{-1},\;p_{j}$ is the allele frequency of the other allele in marker j and M defined in Section 2.1 (Bernal Rubio *et al.*, 2016). This kind of longitudinal analysis was recently performed by Campbell *et al.* (2019) who first estimated the breeding values with a RRM and then used the back-transformation to solve for the SNP effects. Results were compared with an alternative single time point analysis and the authors found that the dynamic analysis recovered more significant associations. The presented model can also be extended by adding fixed environmental effects that affect the phenotype values. This could further reduce the estimation error. Another strategy is to apply a two-stage pre-correction similar to He *et al.* (2016). This means that a linear regression model is first fitted to estimate the fixed effect coefficients and the residuals of that model are then used as phenotype values to estimate the variance components in our LMM.

Further extension would be to consider non-Gaussian longitudinal phenotypes, e.g. binary or count data. In case of binary data, an extra latent-trait layer can be added to the model (Albert and Chib, 1993; Felsenstein, 2005; Kärkkäinen and Sillanpää, 2013). In latent-trait modelling, the binary phenotype can be modelled by considering an underlying hypothetical normally distributed latent-trait variable which gives rise to the binary trait at the observed layer. If the latent-trait variable is smaller than the pre-determined threshold, binary trait at the observed layer obtains the value zero and otherwise it obtains the value one.

One future extension is also to consider multiple longitudinal quantitative traits simultaneously. Some models and methods have been presented for this purpose—to explain the variation of more than one trait simultaneously over time (Oliveira *et al.*, 2019; Sung *et al.*, 2016).

To conclude, we presented a new tuning-free method for estimating dynamic heritability using a Bayesian LMM and GP priors. To estimate the parameters in the model, we use MCMC which makes the uncertainty quantification straightforward. Our results clearly illustrate that joint modelling of the data of all time points reduces the uncertainty in the estimates compared with independent modelling.

## Supplementary Material

btaa199_Supplementary_Data
