# Supplementary material for: Estimation of dynamic SNP-heritability with Bayesian Gaussian process models
Source: Bioinformatics. 2020 Mar 18;36(12):3795–802. doi: 10.1093/bioinformatics/btaa199 (PMC7672693; doi:10.1093/bioinformatics/btaa199)
Supplement: btaa199_Supplementary_Data [file btaa199_supplementary_data.pdf]

Estimation of dynamic SNP-heritability with Bayesian Gaussian process models

Supplementary material

1 Sparse approximation of the Matérn covariance function

The covariance function of a discrete time Gaussian process is represented through a covariance matrix $\boldsymbol{C}$ that has a Cholesky decomposition $\boldsymbol{L}\boldsymbol{L}'$. In the joint method, a square root of the covariance matrix (for example the Cholesky factor $\boldsymbol{L}$) is needed to dewhiten the proposal vector $\boldsymbol{w}$. Generally this is done as $\boldsymbol{x} = \boldsymbol{L}\boldsymbol{w}$, where $\boldsymbol{x}$ is the dewhitened proposal vector. Obtaining the Cholesky factor of a matrix is time consuming in general, so we use a sparse approximation for its inverse. Specifically, the approximate covariance matrix can be expressed as $\boldsymbol{C} \approx (\boldsymbol{H}'\boldsymbol{H})^{-1}$, where $\boldsymbol{H}$ is a sparse matrix that is defined in detail below. To dewhiten $\boldsymbol{w}$, we need to solve $\boldsymbol{x}$ from the system of equations

$$\boldsymbol{H}\boldsymbol{x} = \boldsymbol{w}, \tag{S1}$$

which yields $\boldsymbol{x} = \boldsymbol{H}^{-1}\boldsymbol{w} \approx \boldsymbol{L}\boldsymbol{w}$. This can be efficiently implemented in the linear algebra library Eigen (Guennebaud et al., 2010).

The matrix $\boldsymbol{H}$ is obtained by applying finite-element method to the stochastic partial differential equation representing the Matérn covariance function. The details can be found in Roininen et al. (2014). Here we just give the results. The Matérn correlation function (magnitude parameter in the covariance function set to 1) is given as

$$C(t, t') = \frac{2^{1-\nu}}{\Gamma(\nu)} \left(\frac{|t - t'|}{\lambda} \right)^{\nu} K_{\nu} \left(\frac{|t - t'|}{\lambda} \right), \tag{S2}$$

where ν is the smoothness parameter and λ is the length scale. When ν is set to 1.5, $\boldsymbol{H}$ is a tridiagonal matrix of the form

$$\frac{1}{\sqrt{4\lambda}} \begin{bmatrix} 1+2\lambda^2 & -\lambda^2 & & & 0 \\ -\lambda^2 & 1+2\lambda^2 & -\lambda^2 & & \\ & \ddots & \ddots & & \\ & -\lambda^2 & 1+2\lambda^2 & -\lambda^2 \\ 0 & & & -\lambda^2 & 1+2\lambda^2 \end{bmatrix}$$

and of the same dimension as $\boldsymbol{C}$. Here we assume that the time points are equidistant with distance 1. We note that this approximation is especially bad in the boundaries of the domain (near the first and the last time point measured). A possible remedy for this is to consider a domain extension which is also discussed by Roininen et al. (2014). This means that instead of using just the T time points, we add $T/4$ time points to the beginning and the end of the domain, yielding $1.5T$ time points in total. Hence, when dewhitening the proposal vector with Eq. (S1), the length of the vector is $1.5T$ but in our calculations (e.g. evaluation of the likelihood) we only use the middle part. This way we avoid producing artefacts at the boundaries. This will increase the computation times a little but in our tests we noticed that this increase is minimal compared to not using the approximation at all. In our tests we noticed that the results with the approximation and the exact method were practically indistinguishable.

2 Algorithms

Algorithm 1 Joint model: parameter estimation

Require: $\boldsymbol{\eta}_G^{(1)}, \boldsymbol{\eta}_E^{(1)}, \log \lambda_G^{(1)}, \log \lambda_E^{(1)}, \log \boldsymbol{\sigma}_G^2 = \boldsymbol{C}_G^{\frac{1}{2}} \boldsymbol{\eta}_G^{(1)}, \log \boldsymbol{\sigma}_E^2 = \boldsymbol{C}_E^{\frac{1}{2}} \boldsymbol{\eta}_E^{(1)}$

1: **for** i = 1:(N_{iter} - 1) **do**
2:     simulate $\boldsymbol{v} \sim \mathcal{N}(\boldsymbol{0}, \boldsymbol{I})$
3:     simulate $u \sim \mathcal{U}(0, 1)$
4:     compute $\kappa = \log p_{\mathcal{N}}(\tilde{\boldsymbol{y}}_c | \tilde{\boldsymbol{K}}) + \log u$
5:     simulate $\rho \sim \mathcal{U}(0, 2\pi)$
6:     set $(\rho_{\min}, \rho_{\max}) = (\rho - 2\pi, \rho)$
7:     compute $\boldsymbol{\eta}'_G = \boldsymbol{\eta}_G^{(i)} \cos \rho + \boldsymbol{v} \sin \rho$
8:     compute $\log \boldsymbol{\sigma}_G^2 = \boldsymbol{C}_G^{\frac{1}{2}} \boldsymbol{\eta}'_G$
9:     update $\tilde{\boldsymbol{K}}$
10:     **if** $\log p_{\mathcal{N}}(\tilde{\boldsymbol{y}}_c | \tilde{\boldsymbol{K}}) > \kappa$ **then**
11:         set $\boldsymbol{\eta}_G^{(i+1)} = \boldsymbol{\eta}'_G$
12:     **else**
13:         **if** $\rho < 0$ **then**
14:             set $\rho_{\min} = \rho$
15:         **else**
16:             set $\rho_{\max} = \rho$
17:         **end if**
18:         simulate $\rho \sim \mathcal{U}(\rho_{\min}, \rho_{\max})$
19:         return to step 7.
20:     **end if**
21:     simulate $\log \lambda'_G \sim \mathcal{N}(\log \lambda_G^{(i)}, s_{\lambda_G})$
22:     compute $\log \boldsymbol{\sigma}_G^2 = \boldsymbol{C}_G^{\frac{1}{2}\prime} \boldsymbol{\eta}_G^{(i+1)}$
23:     compute $\alpha_{\lambda_G} = \min \left\{ 1, \frac{p_{\mathcal{N}}(\tilde{\boldsymbol{y}}_c | \tilde{\boldsymbol{K}}') p_{\mathcal{N}}(\log \lambda'_G | \mu_\lambda, \zeta_\lambda^2)}{p_{\mathcal{N}}(\tilde{\boldsymbol{y}}_c | \tilde{\boldsymbol{K}}) p_{\mathcal{N}}(\log \lambda_G^{(i)} | \mu_\lambda, \zeta_\lambda^2)} \right\}$
24:     with probability α_{λ_G} set $\log \lambda_G^{(i+1)} = \log \lambda'_G$, $\tilde{\boldsymbol{K}} = \tilde{\boldsymbol{K}}'$ and $\boldsymbol{C}_G^{\frac{1}{2}} = \boldsymbol{C}_G^{\frac{1}{2}\prime}$ else set $\log \lambda_G^{(i+1)} = \log \lambda_G^{(i)}$

Algorithm 1 (continued)

25: simulate $\boldsymbol{v} \sim \mathcal{N}(\boldsymbol{0}, \boldsymbol{I})$
26: simulate $u \sim \mathcal{U}(0, 1)$
27: compute $\kappa = \log p_{\mathcal{N}}(\tilde{\boldsymbol{y}}_c | \tilde{\boldsymbol{K}}) + \log u$
28: simulate $\rho \sim \mathcal{U}(0, 2\pi)$
29: set $(\rho_{\min}, \rho_{\max}) = (\rho - 2\pi, \rho)$
30: compute $\boldsymbol{\eta}'_E = \boldsymbol{\eta}_E^{(i)} \cos\rho + \boldsymbol{v} \sin\rho$
31: compute $\log \boldsymbol{\sigma}_E^2 = \boldsymbol{C}_E^{\frac{1}{2}} \boldsymbol{\eta}'_E$
32: update $\tilde{\boldsymbol{K}}$
33: **if** $\log p_{\mathcal{N}}(\tilde{\boldsymbol{y}}_c | \tilde{\boldsymbol{K}}) > \kappa$ **then**
34:     set $\boldsymbol{\eta}_E^{(i+1)} = \boldsymbol{\eta}'_E$
35: **else**
36:     **if** $\rho < 0$ **then**
37:         set $\rho_{\min} = \rho$
38:     **else**
39:         set $\rho_{\max} = \rho$
40:     **end if**
41:     simulate $\rho \sim \mathcal{U}(\rho_{\min}, \rho_{\max})$
42:     return to step 30.
43: **end if**
44: simulate $\log \lambda'_E \sim \mathcal{N}(\log \lambda_E^{(i)}, s_{\lambda_E})$
45: compute $\log \boldsymbol{\sigma}_E^2 = \boldsymbol{C}_E^{\frac{1}{2}\prime} \boldsymbol{\eta}_E^{(i+1)}$
46: compute $\alpha_{\lambda_E} = \min \left\{ 1, \frac{p_{\mathcal{N}}(\tilde{\boldsymbol{y}}_c | \tilde{\boldsymbol{K}}') p_{\mathcal{N}}(\log \lambda'_E | \mu_\lambda, \zeta_\lambda^2)}{p_{\mathcal{N}}(\tilde{\boldsymbol{y}}_c | \tilde{\boldsymbol{K}}) p_{\mathcal{N}}(\log \lambda_E^{(i)} | \mu_\lambda, \zeta_\lambda^2)} \right\}$
47: with probability α_{λ_E} set $\log \lambda_E^{(i+1)} = \log \lambda'_E$, $\tilde{\boldsymbol{K}} = \tilde{\boldsymbol{K}}'$ and $\boldsymbol{C}_E^{\frac{1}{2}} = \boldsymbol{C}_E^{\frac{1}{2}\prime}$ else set $\log \lambda_E^{(i+1)} = \log \lambda_E^{(i)}$
48: **end for**

Algorithm 2 Two-stage method: estimation of variance components at stage 1 and time t

Require: $\log \sigma_E^2(t)^{(1)}, \log \sigma_G^2(t)^{(1)}$

for i = 1:(N_{iter} - 1) **do**

  simulate $\log \sigma_E^2(t)' \sim \mathcal{N}(\log \sigma_E^2(t)^{(i)}, s_{\sigma_E^2(t)})$

  compute $\alpha_{\sigma_E^2(t)} = \min \left\{ 1, \frac{p_{\mathcal{N}}(\boldsymbol{y}_c(t)|\boldsymbol{K}(t)')p_{\mathcal{N}}(\log \sigma_E^2(t)'|0,1)}{p_{\mathcal{N}}(\boldsymbol{y}_c(t)|\boldsymbol{K}(t)^{(i)})p_{\mathcal{N}}(\log \sigma_E^2(t)^{(i)}|0,1)} \right\}$

  with probability $\alpha_{\sigma_E^2(t)}$ set $\log \sigma_E^2(t)^{(i+1)} = \log \sigma_E^2(t)'$, else set $\log \sigma_E^2(t)^{(i+1)} = \log \sigma_E^2(t)^{(i)}$

  adapt $s_{\sigma_E^2(t)}$

  simulate $\log \sigma_G^2(t)' \sim \mathcal{N}(\log \sigma_G^2(t)^{(i)}, s_{\sigma_G^2(t)})$

  compute $\alpha_{\sigma_G^2(t)} = \min \left\{ 1, \frac{p_{\mathcal{N}}(\boldsymbol{y}_c(t)|\boldsymbol{K}(t)')p_{\mathcal{N}}(\log \sigma_G^2(t)'|0,1)}{p_{\mathcal{N}}(\boldsymbol{y}_c(t)|\boldsymbol{K}(t)^{(i)})p_{\mathcal{N}}(\log \sigma_G^2(t)^{(i)}|0,1)} \right\}$

  with probability $\alpha_{\sigma_G^2(t)}$ set $\log \sigma_G^2(t)^{(i+1)} = \log \sigma_G^2(t)'$, else set $\log \sigma_G^2(t)^{(i+1)} = \log \sigma_G^2(t)^{(i)}$

  adapt $s_{\sigma_G^2(t)}$

end for

Algorithm 3 Two-stage method: estimation of smoothing model parameters at stage 2

Require: $\log \gamma^{2(1)}, \log \lambda^{(1)}$

for i = 1:(N_{iter} - 1) **do**

  simulate $\log \gamma^{2'} \sim \mathcal{N}(\log \gamma^{2(i)}, s_{\gamma^2})$

  compute $\alpha_{\gamma^2} = \min \left\{ 1, \frac{p_{\mathcal{N}}(\boldsymbol{y}|\boldsymbol{C}_{\lambda^{(i)}} + \gamma^{2'}\boldsymbol{I})p_{\mathcal{N}}(\log \gamma^{2'}|0,1000)}{p_{\mathcal{N}}(\boldsymbol{y}|\boldsymbol{C}_{\lambda^{(i)}} + \gamma^{2(i)}\boldsymbol{I})p_{\mathcal{N}}(\log \gamma^{2(i)}|0,1000)} \right\}$

  with probability α_{γ^2} set $\log \gamma^{2(i+1)} = \log \gamma^{2'}$, else set $\log \gamma^{2(i+1)} = \log \gamma^{2(i)}$

  adapt s_{γ^2}

  simulate $\log \lambda' \sim \mathcal{N}(\log \lambda^{(i)}, s_\lambda)$

  compute $\alpha_\lambda = \min \left\{ 1, \frac{p_{\mathcal{N}}(\boldsymbol{y}|\boldsymbol{C}_{\lambda'} + \gamma^{2(i+1)}\boldsymbol{I})p_{\mathcal{N}}(\log \lambda'|\mu_\lambda, \zeta_\lambda^2)}{p_{\mathcal{N}}(\boldsymbol{y}|\boldsymbol{C}_{\lambda^{(i)}} \gamma^{2(i+1)}\boldsymbol{I})p_{\mathcal{N}}(\log \lambda^{(i)}|\mu_\lambda, \zeta_\lambda^2)} \right\}$

  with probability α_λ set $\log \lambda^{(i+1)} = \log \lambda'$, else set $\log \lambda^{(i+1)} = \log \lambda^{(i)}$

  adapt s_{λ^2}

end for

3 Traceplots for the joint model

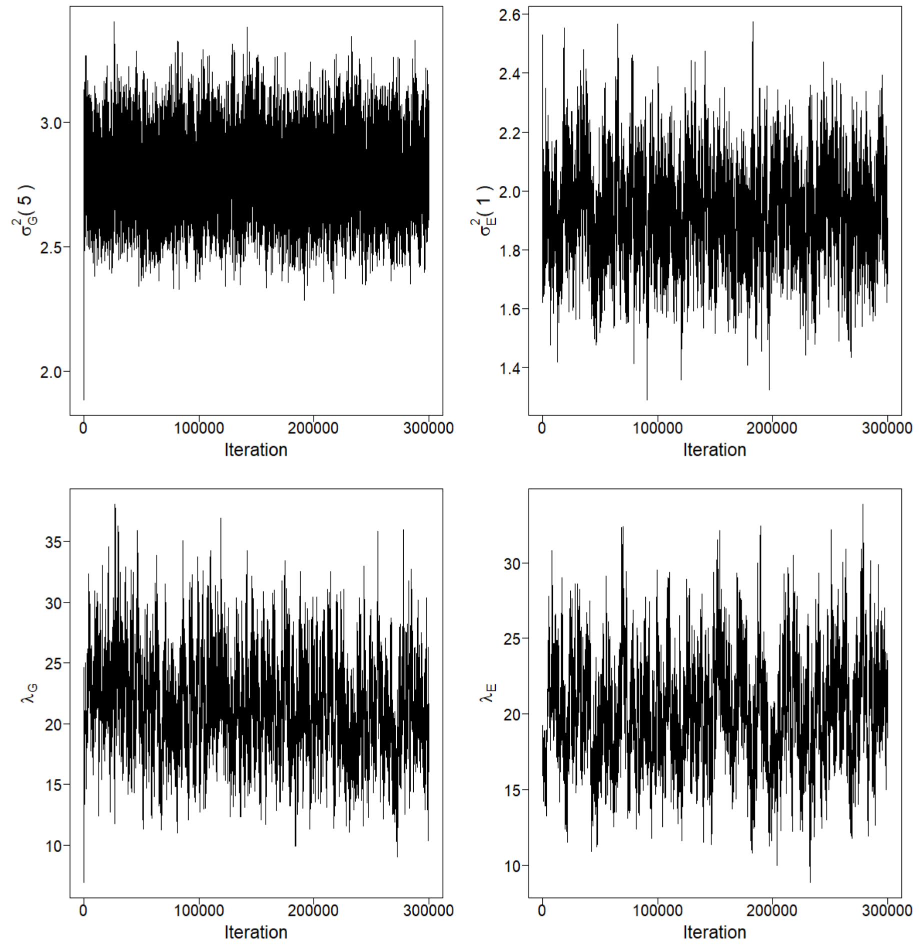

Figure S1: Some traceplots of parameters in the joint model for the simulated data: (top left) variance component parameter with the highest effective sample size (ESS), (top right) variance component parameter with the lowest ESS, (bottom left) length scale of the genetic variance process, (bottom right) length scale of the environmental variance process.

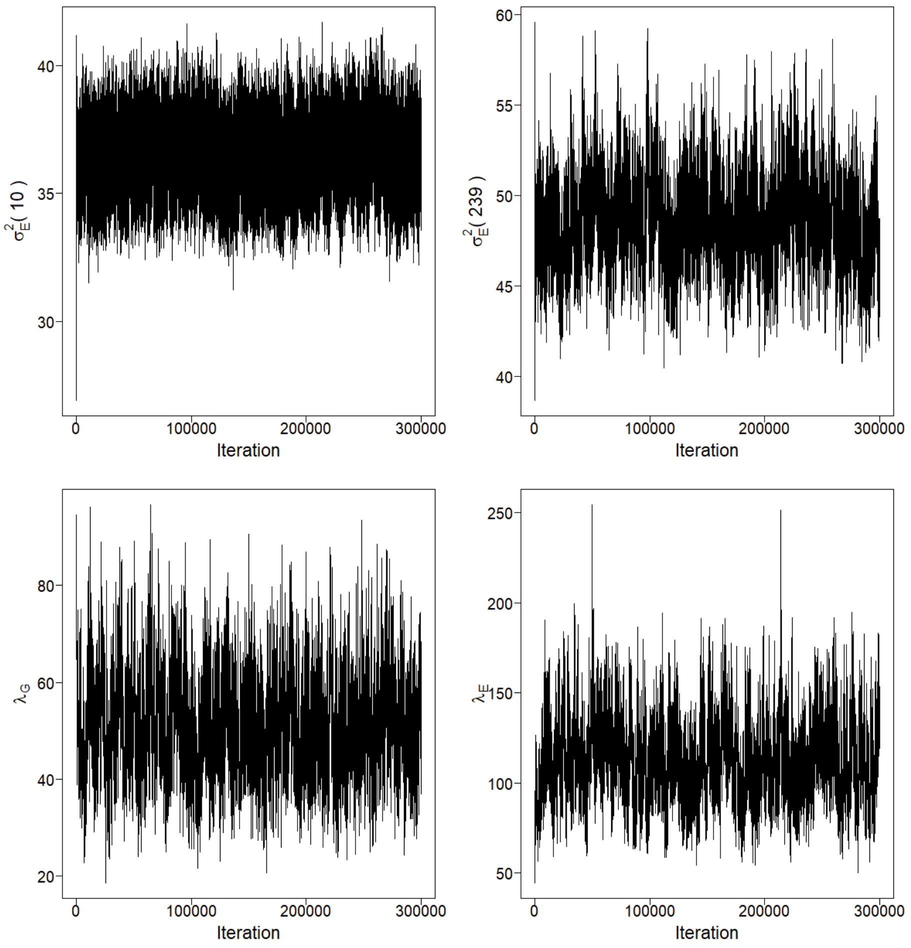

Figure S2: Some traceplots of parameters in the joint model for the *Arabidopsis thaliana* data: (top left) variance component parameter with the highest effective sample size (ESS), (top right) variance component parameter with the lowest ESS, (bottom left) length scale of the genetic variance process, (bottom right) length scale of the environmental variance process.

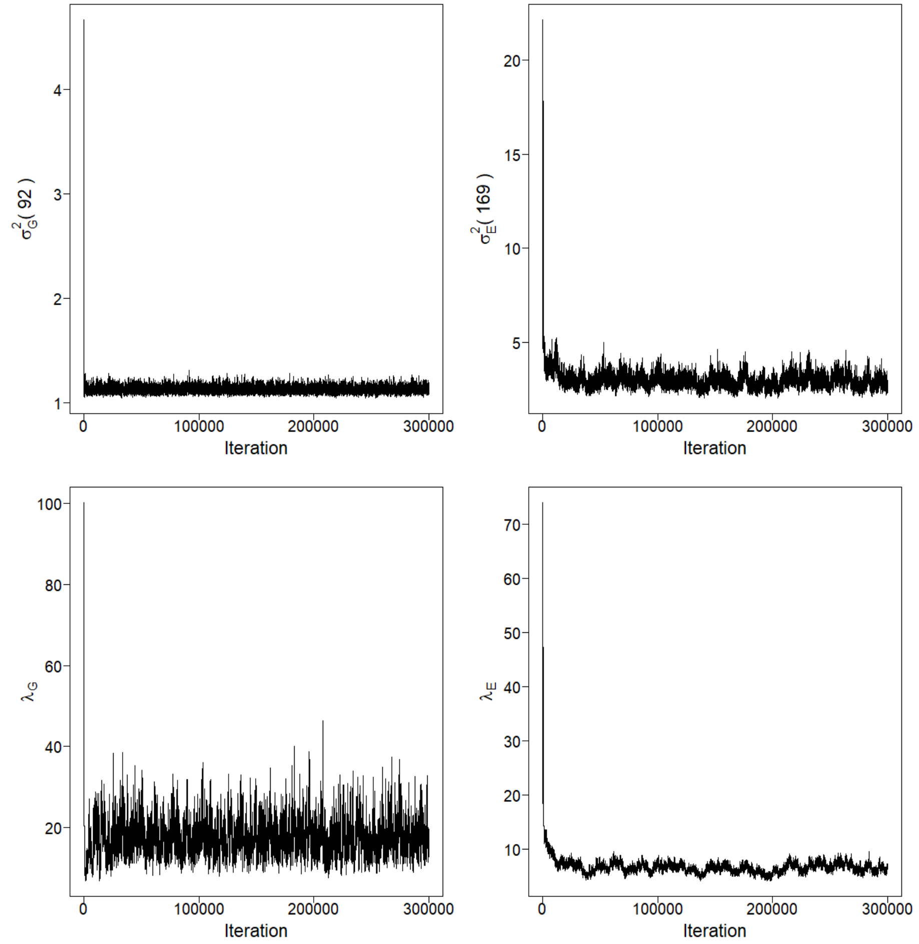

Figure S3: Some traceplots of parameters in the joint model for the mouse activity data: (top left) variance component parameter with the highest effective sample size (ESS), (top right) variance component parameter with the lowest ESS, (bottom left) length scale of the genetic variance process, (bottom right) length scale of the environmental variance process.

4 Comparison with ACEt

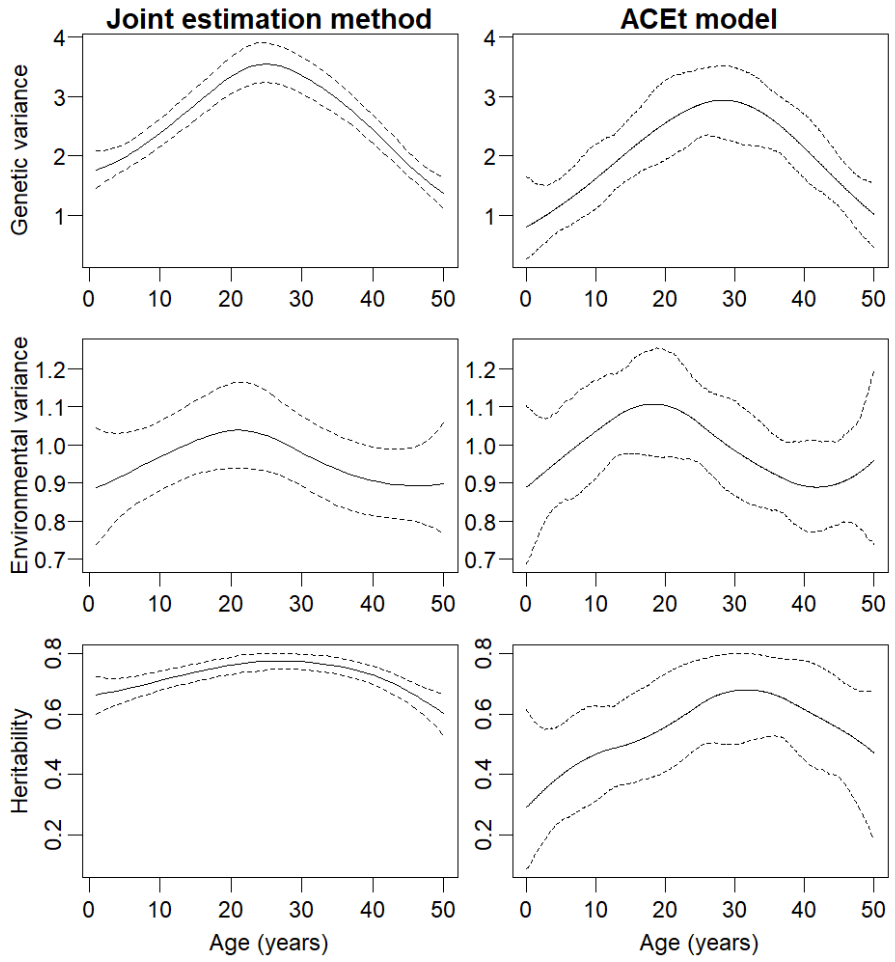

Figure S4: Comparison of the results from the joint estimation method and ACEt on twin data. Posterior means for the joint method and maximum likelihood estimates for ACEt are drawn with solid lines and 95 % credible intervals for the joint method and 95% confidence intervals for ACEt with dashed lines.

5 Computation times

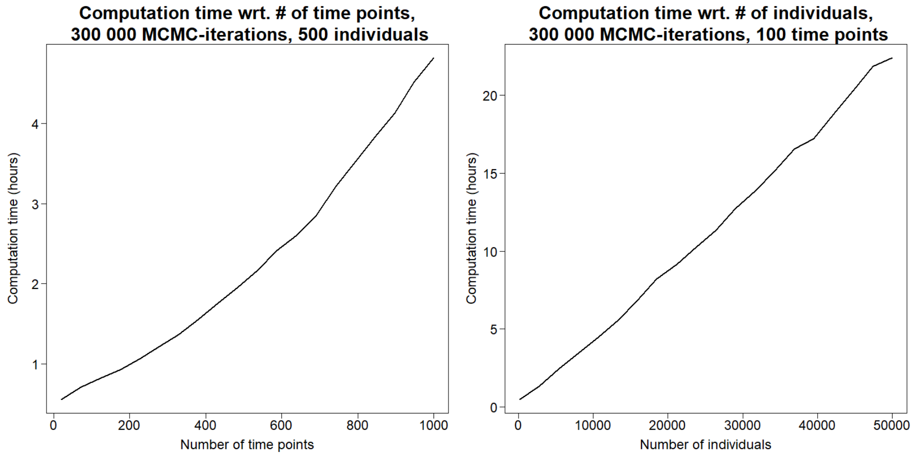

Figure S5: Empirical computation times of the joint method with respect to the number of time points and individuals.

6 Performance comparison with the joint method and random regression model (RRM)

The accuracies of the joint method and RRM as implemented with MTG2 were compared by simulating data and computing mean squared errors (MSE) between the estimated variances and the ground truths. We simulated ten datasets with 1000 individuals and 50 time points with the variances $\sigma_G^2(t) = \cos t + 2$ and $\sigma_E^2(t) = \sin t + 2$, where the time points were equidistant in the interval $[0, 2\pi]$ hours. The relationship matrix was created by first simulating a 1000×1000 matrix $\boldsymbol{S}$ where each element is standard normally distributed. The relationship matrix was then computed as $\boldsymbol{G} = \boldsymbol{S}\boldsymbol{S}^T/1000 + 0.1\boldsymbol{I}$. A small number was added to the diagonal to make the matrix positive definite. To simulate realistic data, the longitudinal dependencies need to also be taken into account. This was done by generating a 50×50 Gaussian process matrix $\boldsymbol{C}$ where the row i and column j intersection was set to $[C]_{ij} = \left(1 + \frac{\sqrt{5}|t_i - t_j|}{(50/3)} + \frac{5(t_i - t_j)^2}{3 \cdot (50/3)^2}\right) \exp\left(-\frac{\sqrt{5}|t_i - t_j|}{(50/3)}\right)$. The genetic and environmental components of the data were simulated from the distributions $\mathcal{N}(\boldsymbol{0}, \boldsymbol{C} \otimes \boldsymbol{G})$ and $\mathcal{N}(\boldsymbol{0}, \boldsymbol{C} \otimes \boldsymbol{I})$, respectively. Finally, the components were scaled with the corresponding variance at each time point and summed. The datasets were analysed with the joint method with 300 000 MCMC-iterations and with RRM using Legendre polynomials of degree 5 to model the genetic and residual covariances. The RRM at time t was defined as

$$\boldsymbol{y}_t = \boldsymbol{1}\alpha_t + \boldsymbol{a}\boldsymbol{\Phi}_t' + \boldsymbol{b}\boldsymbol{\Phi}_t' + \boldsymbol{\epsilon}_t, \tag{S3}$$

where α_t is the overall mean at time t, $\boldsymbol{a} \in \mathbb{R}^{N\times 6}$ contains the genetic random regression coefficients, $\boldsymbol{b} \in \mathbb{R}^{N\times 6}$ contains the residual random regression coefficients, $\boldsymbol{\Phi}_t$ is the tth row of a $T \times 6$ matrix that contains the Legendre polynomials up to degree 5 evaluated at the T measurement points and $\boldsymbol{\epsilon}_t$ are the residuals at time t which are assumed to be independently normally distributed but with different variances across time. Here $\boldsymbol{b\Phi}_t'$ and $\boldsymbol{\epsilon}_t$ can be interpreted as the dependent and independent residual, respectively. To fit the model we used the software MTG2 (Lee and van der Werf, 2016). The software only supports frequentistic statistical inference. In particular, it uses the average information (AI-REML) algorithm to maximise the likelihood function.

The MSE for the joint method was computed from the posterior mean. The MSE for the estimated environmental variance (permanent environmental variance + residual variance), genetic variance, and heritability for both, the joint method and MTG2, can be found in Table S1. Additionally, we present averaged estimates from all of the datasets in Fig. S6.

Additionally, we fitted a Bayesian version of the model (S3) using BLUPF90 family of programs (Misztal et al., 2002) and in particular GIBBS2F90. This allows us to compare the differences of the uncertainties of the variance components between RRM and the joint model. The program uses inverse Wishart distribution as a prior for the covariance matrices of the random regression coefficients. To assign the priors, we first ran the frequentistic inference using MTG2 and used the results from that analysis as prior scale matrices for the inverse Wishart distribution. The degree of belief was set to 10 for both the genetic and residual covariance matrices. The degree of belief for the independent residual was set to 1. The results from this analysis are presented in Fig. S7. Due to long computation times of GIBBSF90 software (over 24 hours) only one dataset was analysed and MSE comparison was infeasible to carry out.

	MSE_E		MSE_G		MSE_{h^2}	
	Joint method	MTG2	Joint method	MTG2	Joint method	MTG2
1	8.0×10^{-3}	1.2×10^{-2}	6.6×10^{-2}	5.2×10^{-2}	1.2×10^{-3}	1.0×10^{-3}
2	8.5×10^{-2}	8.6×10^{-2}	6.0×10^{-2}	7.8×10^{-2}	3.5×10^{-3}	4.0×10^{-3}
3	5.6×10^{-2}	4.8×10^{-2}	1.6×10^{-1}	1.8×10^{-1}	2.4×10^{-3}	2.1×10^{-3}
4	6.5×10^{-2}	5.2×10^{-2}	1.2×10^{-2}	2.5×10^{-2}	1.5×10^{-3}	2.0×10^{-3}
5	1.1×10^{-2}	2.5×10^{-2}	6.3×10^{-3}	2.0×10^{-2}	3.0×10^{-4}	9.5×10^{-4}
6	4.1×10^{-2}	3.7×10^{-2}	9.3×10^{-2}	1.1×10^{-1}	1.7×10^{-3}	2.3×10^{-3}
7	1.3×10^{-1}	1.5×10^{-1}	8.6×10^{-2}	1.2×10^{-1}	4.0×10^{-3}	5.6×10^{-3}
8	1.1×10^{-2}	1.6×10^{-2}	3.4×10^{-2}	6.1×10^{-2}	4.7×10^{-4}	1.4×10^{-3}
9	4.3×10^{-2}	5.1×10^{-2}	2.9×10^{-2}	4.1×10^{-2}	1.2×10^{-3}	1.6×10^{-3}
10	4.0×10^{-2}	5.1×10^{-2}	2.8×10^{-2}	5.1×10^{-2}	1.4×10^{-3}	2.2×10^{-3}
Average	4.9×10^{-2}	5.2×10^{-2}	5.7×10^{-2}	7.4×10^{-2}	1.8×10^{-3}	2.3×10^{-3}
SD	3.8×10^{-2}	4.0×10^{-2}	4.7×10^{-2}	5.0×10^{-2}	1.2×10^{-3}	1.4×10^{-3}

Table S1: Mean squared error (MSE) calculated from the estimates of environmental variance (E), genetic variance (G) and heritability (h^2) of the joint method and random regression model implemented with MTG2 for the ten different simulated datasets.

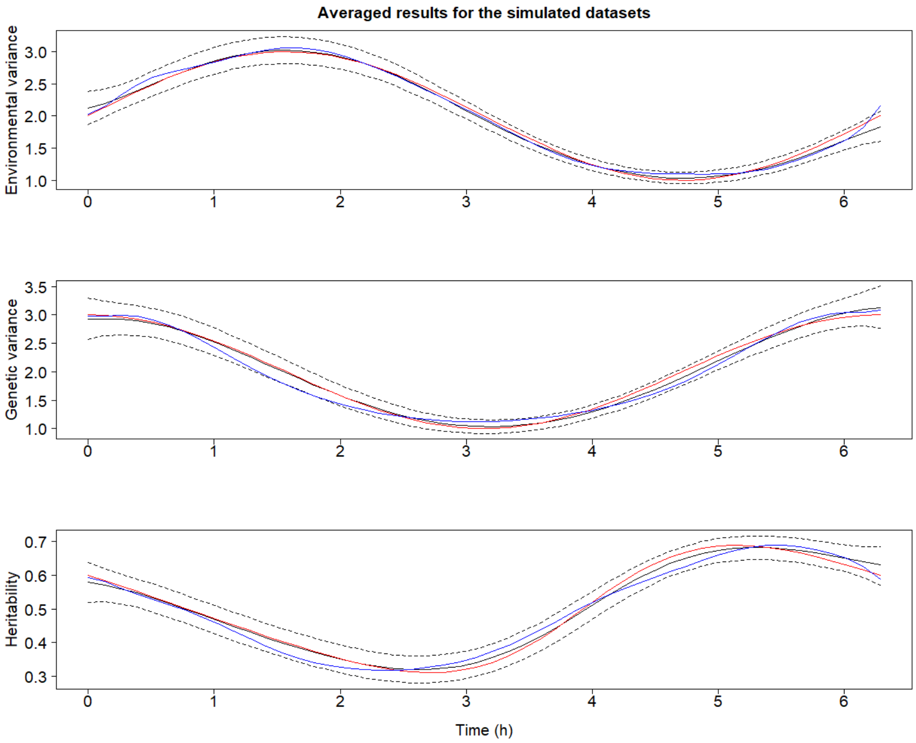

Figure S6: Averaged results from the ten simulated datasets for the joint method (black line) and MTG2 (blue line). The averaged 95% credible intervals of the joint method are drawn with dashed lines and the ground truth with red line.

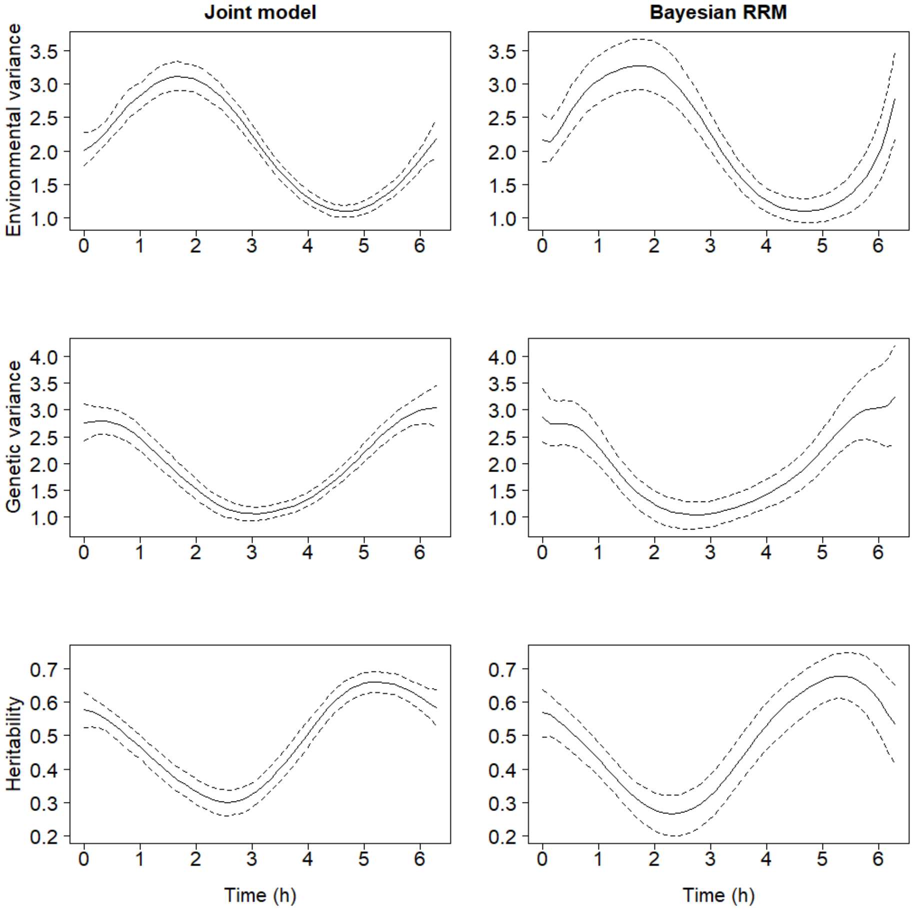

Figure S7: Comparison of the joint model and Bayesian RRM implemented with GIBBS2F90. Posterior means are drawn with solid lines and 95% credible intervals with dashed lines.

References

Guennebaud, G., Jacob, B., et al. (2010). Eigen v3. http://eigen.tuxfamily.org.

Lee, S. and van der Werf, J. (2016). MTG2: An efficient algorithm for multivariate linear mixed model analysis based on genomic information. *Bioinformatics*, 32:1420–1422.

Misztal, I., Tsuruta, S., Strabel, T., Auvray, B., Druet, T., and Lee, D. (2002). BLUPF90 and related programs (BGF90). *Proceedings of the 7th world congress on genetics applied to livestock production*, 33:743–744.

Roininen, L., Huttunen, J., and Lasanen, S. (2014). Whittle-Matérn priors for Bayesian statistical inversion with applications in electrical impedance tomography. *Inverse Problems and Imaging*, 8:561 – 586.
